# Sustaining community self-help groups beyond donor support: lessons from a qualitative study of self-help groups, including persons affected by leprosy and disability in rural India

**DOI:** 10.1136/bmjopen-2025-110417

**Published:** 2026-01-09

**Authors:** Joydeepa Darlong, Mythily VS Charles, Onaedo Ilozumba, Karthikeyan Govindasamy, Anjali Shrivastva, Sopna Choudhury, Jo Sartori, Antje Lindenmeyer, Richard J Lilford, Frances Griffiths

**Affiliations:** 1 The Leprosy Mission Trust India, New Delhi, India; 2 Department of Applied Health, University of Birmingham, Birmingham, UK; 3 Applied health, University of Birmingham - Edgbaston Campus, Birmingham, UK; 4 Warwick Centre for Global Health, University of Warwick, Coventry, UK

**Keywords:** QUALITATIVE RESEARCH, Public health, Social Support, INFECTIOUS DISEASES, Disabled Persons, Self-Management

## Abstract

**Abstract:**

**Introduction:**

Leprosy remains a significant public health challenge in many low and middle-income countries, including India. People affected by leprosy face multifaceted challenges: physical, psychological, social and economic. In response, donors support self-help groups (SHGs) to improve health, social integration and economic circumstances for marginalised people, including those with leprosy. This study aims to assess the sustainability of SHGs in India after the withdrawal of donor support by examining whether they remain functional and exploring the key factors, barriers and facilitators that influence their long-term social and economic viability.

**Objectives:**

To examine the functionality of SHGs after withdrawal of donor support, and to explore the factors, barriers and facilitators influencing their long-term social and economic sustainability.

**Methods:**

Using qualitative methods, we conducted semistructured interviews with 40 key informants associated with five SHGs formed under the Self-Help Community Development Project implemented in an endemic state of India and funded by The Leprosy Mission Trust India.

**Study design:**

It was an exploratory qualitative study using interviews with SHG members and key informants, situated within the self-help community-based project.

**Results:**

While some SHGs demonstrated resilience and adaptability, others faced challenges such as internal discord, loss of members to migration and lack of access to government schemes. Thematic analysis revealed key drivers and barriers to sustainability and realising the benefits of SHGs, highlighting variations in leadership, governance, economic performance and social engagement across groups.

**Discussion and conclusion:**

SHGs are often sustained after the funding and managerial donor support have been withdrawn. The findings emphasise the importance of strong leadership, community support and external facilitation in sustaining SHGs and enhancing their impact on marginalised populations. This study contributes to understanding the role of SHGs in addressing the socioeconomic challenges faced by individuals affected by leprosy and offers insights for improving their long-term viability.

**STRENGTHS AND LIMITATIONS OF THIS STUDY**

- This is one of the first studies to examine the sustainability of self-help groups (SHGs) formed among persons affected by leprosy and disability in India.
- An exploratory qualitative design allowed in-depth insights into the experiences and perspectives of SHG members and community stakeholders.
- Triangulation of perspectives from SHG members, leaders and external stakeholders increased the credibility of findings.
- As a qualitative study, findings provide transferable lessons for similar community-based programmes.
- The study was conducted in a single state in India, which may limit the applicability of results to other settings.

## Layman’s summary

 Self-help groups (SHGs) in India are small groups of mostly women who come together to save money, lend to each other and take up small income-generating activities. They are often supported by outside organisations for a few years, with the hope that they will continue on their own afterwards. However, whether SHGs remain active and useful after external support ends is not always clear.

We explored this question by talking to SHG members and community stakeholders in Chhattisgarh, India, more than 2 years after project funding ended. We studied five SHGs, of which three were still active, one was partly functional and one had stopped working. The unique feature about these SHGs was that the members were heterogeneous having included women affected by leprosy and/or disability.

Our findings showed that sustainability depends on three main drivers: leadership and governance, economic performance and social performance. SHGs with transparent and inclusive leaders, flexible rules and regular meetings were more likely to continue. Intralending—where members borrow from group savings at low interest—was the strongest economic activity keeping groups together. Groups also tried small livelihood projects, but these were often risky and less reliable. Social benefits were just as important as financial ones. SHGs reduced stigma related to leprosy and disability, supported families during crises and even campaigned on community issues like sanitation and alcohol use.

SHGs can sustain themselves beyond outside support if leadership is strong, governance is fair and members feel socially empowered. To help SHGs thrive, programmes should invest in leadership training, flexible governance, access to government schemes and better support for economic activities.

## Introduction

### Background

Leprosy remains a public health problem in many low- and middle-income countries (LMICs)^1 2^ and even though India met the WHO’s criteria for eliminating leprosy as a public health problem in 2005, leprosy continues to be endemic in some districts.^3^ The National Leprosy Eradication Programme reported that the state of Chhattisgarh was yet to achieve elimination with a high leprosy prevalence rate of 2.3/10 000, which is the highest in India with a high percentage of Grade 2 Disabilities in the endemic districts.^4 5^

People affected by leprosy, especially those living in rural areas, face physical, psychological, social and economic challenges. This is due to the chronic and disabling nature of the disease, as well as ignorance and misinformation amidst some communities. Despite years of effort from both the government and NGOs (non governmental organizations), people affected by leprosy continue to experience significant impacts of the disease on all aspects of life, creating the need for sustainable medical and social interventions targeted towards creating a better quality of life for these individuals. 6,9

People with leprosy neuropathy have relied heavily on self-care interventions to improve health outcomes specially by reducing ulcer prevalence. These interventions use groups to teach practices that individuals affected by leprosy undertake by themselves to improve their health outcomes and manage the effects of the disease. This usually involves education on self-care, disability and wound care management, and provision of footwear.

SHGs have become important community-based structures supporting health, livelihoods and social empowerment in LMICs, in contexts of filariasis,^10^ neglected tropical diseases, disabilities^11 12^ and leprosy.^13^ These groups are typically formed by individuals facing similar conditions who come together to provide mutual support, encouragement and assistance to one another. Since their formal inception in 1976, SHGs have emerged as grassroots interventions to empower individuals and provide a platform to work together, support each other and address shared challenges effectively.^14^ SHGs include self-employment and income-generating opportunities, create easier access to banks for microcredit and other subsidies, build capacity by training, promote intralending and savings, and serve as a platform for education and social activism.^13 15^

**Figure 1:**
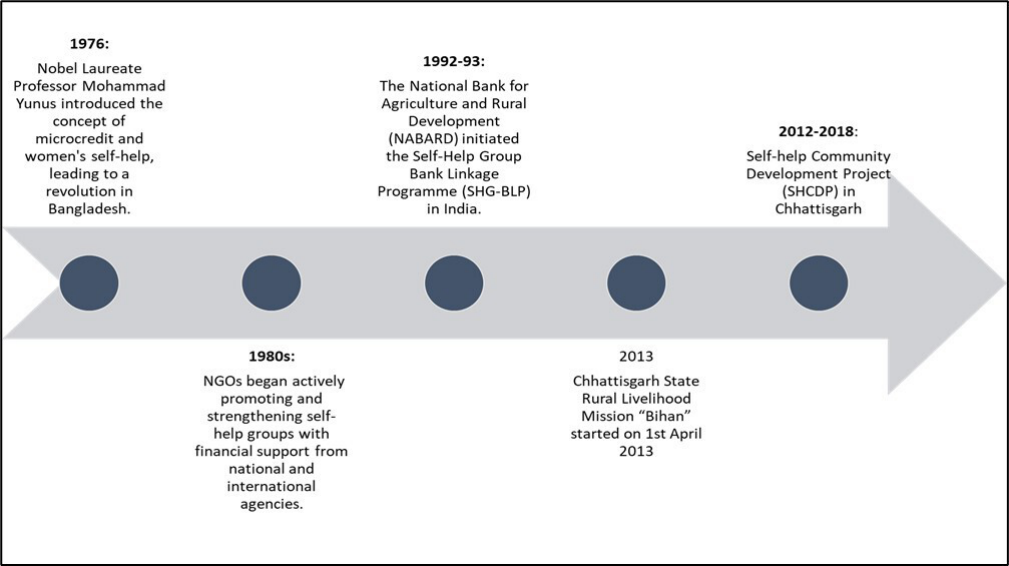
Timeline of SHGs.

In India, there are an estimated 9 million SHGs with 200 million members that function with a goal of economic empowerment. The evolution of the SHG movement progressed through distinct phases, each marked by significant developments and changes in approach. The evolution and India’s role in the programme is shown in figure 1.

SHGs were initially instituted and promoted by non-governmental organisations such as The Leprosy Mission International or Self-Employed Women’s Association,^16^,^18^ who played a crucial role in mobilising communities, creating awareness and facilitating group formation. The National Bank for Agriculture and Rural Development (NABARD) played a key role in introducing the SHG-bank linkage model (BLM) where SHGs were connected with formal institutions to provide financial access to the members. However, some SHGs chose to carry out savings and intralending activities without accessing more formal microcredit for business ventures. State governments became actively involved in the SHG movement, recognising its potential for socioeconomic development. This helped with the formulation of policies and programmes to support and enhance the impact of SHGs.

The SHG-BLM expanded, achieving large-scale success across regions, thus making SHGs recognisable as reliable entities for financial institutions and so facilitating increased credit flow. SHG federations started to emerge, bringing together multiple SHGs under a common umbrella, which provided a platform for collaboration, resource sharing and collective decision-making. They gained recognition as effective agents from mainstream agencies, including government departments, and their role expanded beyond microfinance to include implementation of various development programmes.^19^,^22^

Recent global evidence demonstrates that peer-support collectives improve chronic disease outcomes, treatment adherence, mental health and reduce disability-related stigma.^23^ Our study expands this knowledge by examining sustainability after donor support withdrawal in mixed-membership SHGs including persons affected by leprosy.

SHGs in India are small, voluntary collectives of 10–20 members—mostly women—registered with local authorities and linked to a bank account, to access government schemes and financial support. Their core activities include regular savings, internal lending at nominal interest rates and microcredit. Many also engage in income-generating ventures such as handicrafts, tailoring, dairy farming and catering. Recognised SHGs benefit from schemes like the National Rural Livelihood Mission and the Midday Meal Scheme, where they may supply food to schools.

SHGs are usually supported for a limited period (3–5 years), with the expectation that they will become self-sustaining or evolve into larger structures such as SHG bank linkages federations,^15^ and cooperatives, or microenterprises.

However, the concept of ‘sustainability’ is interpreted differently across studies. Some define it as long-term financial viability independent of promoting institutions or federations,^24^,^26^ without reliance on promoting institutions or higher-level structures such as federations^27^ while others consider sustained linkages with financial institutions as a key benchmark.^26^ Still others associate sustainability with group structure and organisation, noting that effective management underpins strong financial and institutional performance.^28 29^

Given this diversity, several authors call for a more comprehensive and grounded assessment of sustainability.^27^,^29^ Since no single framework or consensus exists,^29 30^ we adopted an exploratory, qualitative approach to examine whether, and in what form, SHGs persist socially and economically once donor support is withdrawn. Our analysis, based on the perspectives of SHG members and other key informants in a community-based project in Chhattisgarh, India, used a working definition of sustainability as the continued active engagement of members in financial and social activities. We sought to identify the factors influencing group functionality and to explore barriers and facilitators of sustainability after external support ended.

## Methods and data collection

### Study design

Exploratory qualitative methodology was applied to collect information through semistructured interviews in order to understand barriers and facilitators which are crucial to sustainability of SHGs. Our study was conducted as part of the National Institute for Health and Care Research and Research and Innovation for Global Health Transformation grant, which aims to enhance care for individuals affected by leprosy and Buruli ulcer.^31^ The Consolidated Criteria for Reporting Qualitative Research (COREQ), a 32-item checklist, was followed to ensure that essential aspects, including the roles of the research team, reflexivity, study design, data analysis and findings, were properly addressed.^32^

### Study context

The study focused on the Self-Help Community Development Project (SHCDP) which was implemented in Chhattisgarh from 2012 to 2018. Chhattisgarh is a state in Central India known for a significant population living in poverty and being affected by leprosy.

**Figure 2:**
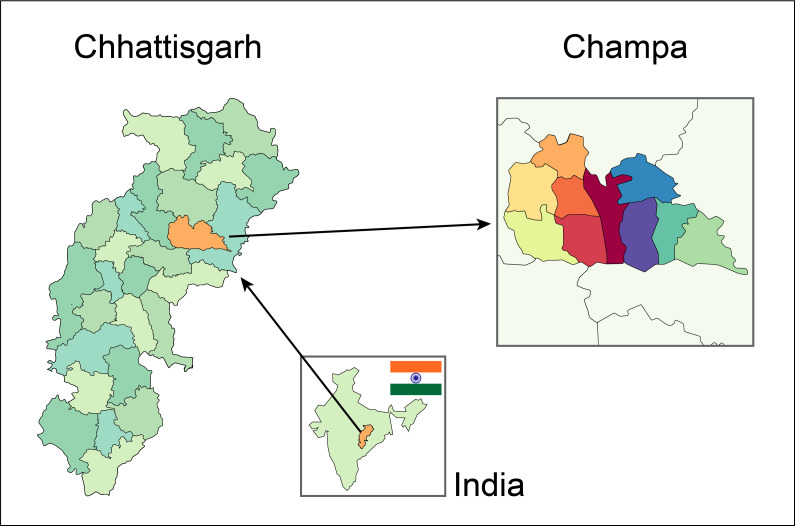
Map of the study site.

The study focused on five panchayats (local government offices) in Nawagarh block, a subdivision of the Champa district, which is largely rural and carries the highest burden of leprosy cases and related complications in Chhattisgarh state (figure 2).

The SHCDP was funded by The Leprosy Mission Trust India(TLMTI), the largest leprosy-focused NGO in India. Edited to The leprosy mission Trust India(TLMTI) had initiated self-care groups among women affected by leprosy to help them manage their disabilities. However, it soon became clear that self-care alone was not enough to address the challenges these women faced; economic deprivation, stigma, exclusion and discrimination also needed to be tackled. In response, pilot SHG projects were implemented in different states, aimed at promoting both health and economic well-being. The SHCDP was unique as it created SHGs with female members from the general population including, but not limited to, women affected by leprosy and disability. It was believed that being a heterogeneous group, more disadvantaged women would benefit through the economic and social initiatives alongside those who were better off, thus reducing the disparity in social status.^33^

### Study participants

From 36 groups that were formed with the support from the SHCDP, we purposively selected five area-based SHGs with at least one member affected by leprosy and/or a disability. To maintain anonymity, we designated these groups as A, B, C, D and E. The SHCDP staff facilitated our introduction to the group members.

**Figure 3:**
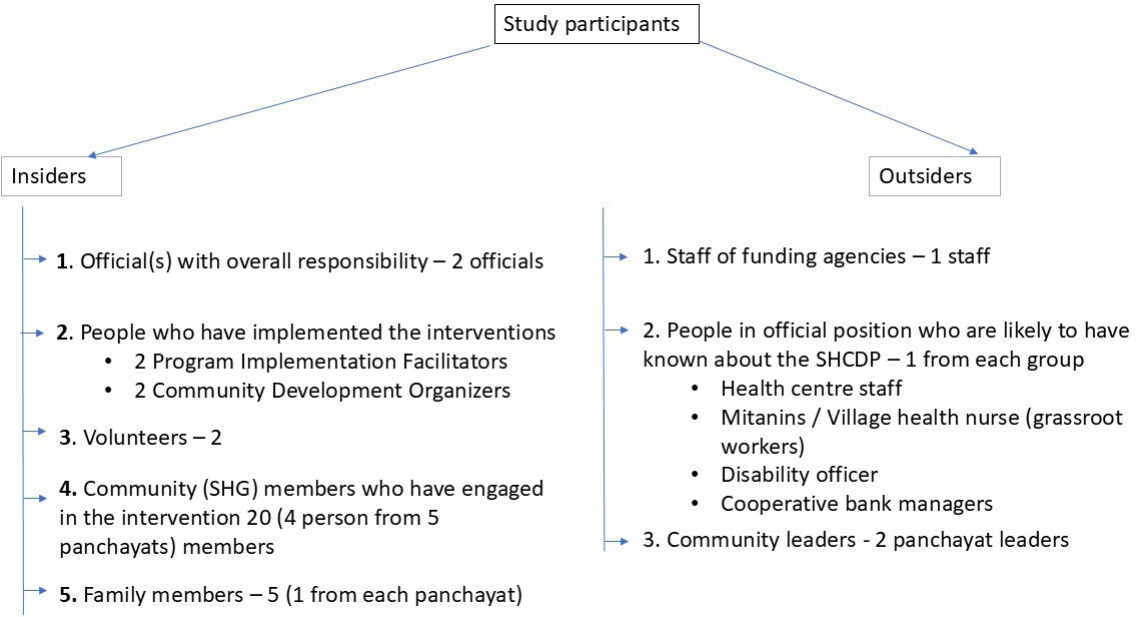
Study Participants

There were two kinds of participants: Insiders (n=33) and Outsiders (n=7) (figure 3). Insiders were individuals directly engaged in the project during its funded period, for example, SGH members and their families and SGHDP staff. Outsiders were those who were more indirectly involved. Researchers were previously unknown to the participants. A purposive sampling technique was used (based on roles such as secretary, president, person affected by leprosy/disability) to select study participants from the SHGs. Snowball sampling and recommendations from board members and project staff were used to identify additional participants.

### Researcher positionality

The research team included clinicians and public-health researchers affiliated with an NGO historically supporting persons affected by leprosy. To reduce potential bias, independent transcription and intercoder reliability checks were undertaken, reflexive discussions were held and COREQ guidelines were followed.

### Data collection

**Figure 4:**
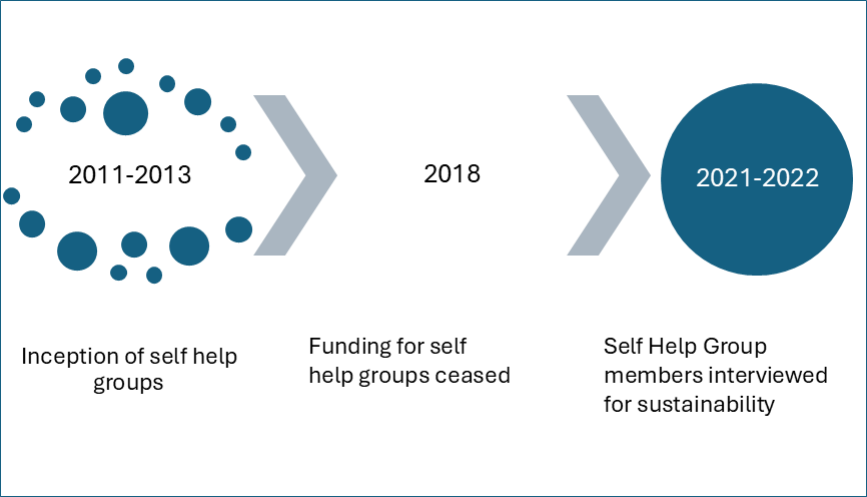
Timeline of self-help groups (SHGs) from inception to time of interview.

Forty semistructured interviews were conducted, after getting familiarised with the participants, lasting between 30 and 60 min. The interviews were conducted by the principal investigator (PI) and co-PI for the study who were trained in qualitative research methods by staff at the University of Birmingham. They were conducted in the community settings till data saturation. Written informed consent was obtained from all participants. The interview guides (see online supplemental appendix) were developed by the research teams, pilot tested and refined. There were no repeat interviews.^34^ Interviews were digitally recorded, translated and transcribed into English by an independent agency and then imported into QSR. The timeline of SHGs from inception to time of interview is shown in figure 4. Data collection for the study took place between March 2021 and December 2022 (ie, 3–4 years after funding ceased).

### Data analysis

The transcripts were thoroughly reviewed to ensure they accurately reflected the study context. This involved repeated readings (AS and MVSC) to gain familiarity with the interview content, followed by the coding and comparison of data. Subsequently, themes were identified, organised and refined. To ensure consistency and conceptual clarity, a coding framework or ‘codebook’, as described by Braun and Clarke^35^ was developed by the lead author (JD) and research associates (MVSC and KG). This codebook was based on a review of three transcripts and team discussions.

The remaining transcripts were then coded by the research associates, with regular meetings to review coding consistency, consult field notes and refine the codebook as necessary (MVSC). Intercoder reliability was also tested using the NVivo software. For the synthesis of the coded data, an NVIVO matrix was developed to provide a thorough structure for the interpretation of the findings (MVSC). Findings were discussed informally with community members for validation.

### Ethics approval

The study received ethical approval from The Leprosy Mission Trust India Ethics Committee (TLMTI EC/S-46) and the University of Birmingham Biomedical and Scientific Research Ethics Committee. Pseudonyms were used for names and places to maintain confidentiality. The recorded file was encoded based on participant number, study site and participant role to ensure anonymity of the participants.

## Results

Study results are presented in two parts: first, a ‘pen portrait’ is provided for each group to create a holistic understanding of their inception and development. Online supplemental table S1 shows the comparison of the profiles of the SHG (Self-Help Group). This is followed by a report of the themes we identified.

### Description of SHGs in the study

#### SHG A: the enterprising group

Members of this group had joined from other existing groups and had experience of being a SHG member for 5–6 years. They also had support from their families. The group’s secretary was a person affected by leprosy and was illiterate. During the funding period, members held monthly meetings, contributed rupees 50 each per month and engaged in intragroup lending at an interest rate of 2%. They received training in livelihood activities and participated in leprosy awareness sessions, which covered the causes of leprosy, its symptoms, modes of transmission, treatment options, the importance of early diagnosis and ways to reduce stigma within the community. While members were somewhat successful in carrying out livelihood activities (eight members were involved in the governmental midday meal scheme), they were very pro-active in social activities (closing down liquor shops, women’s rights, leprosy awareness, etc) and charity work. When the promoter organisation support ended in 2018, they had 19 members. This meant that the group was ineligible for government schemes as their numbers were more than stipulated; however, they did not want to remove members as they felt larger groups could make more profit. Group members continued to deposit money; they appeared united and were willing to work if further opportunities arose.

#### SHG B: the functional group

After observing other functional groups in their village, women expressed a desire to join one themselves. When the NGO introduced the idea of forming a SHG, they embraced the opportunity and established a group. Initially, it comprised 20 members, but over time, the number reduced to 10 due to family pressures and internal disagreements. Despite these challenges, the group has remained active and has been functioning since 2016.

The secretary appeared to be more educated than the leader and managed most of the internal activities. The group leader was chosen for her ability to travel around. They had a supportive family, appeared united and took decisions together. Some internal lending was done, and they deposited their savings in the bank and distributed it with the interest gained among themselves at the end of the year. They have had livelihood (economic) training and ran two grocery shops; they claimed they had not got any government schemes because their village president was partial to other groups. The group did not get involved in any social activities. After the donor organisation support ended, for the initial 2 years they were depositing rupees 50 on the third of every month, and then they increased it to rupees 100. Some wanted to continue just savings, but a few wanted to do some economic activities.

#### SHG C: the inactive group

The group started in 2014 and discontinued after running 4–5 years. It had 13 members, most of whom were related to each other. Some members said they did not have any livelihood training and they were never involved in any livelihood activities though they were registered and had a bank account. Members felt that the sarpanch (village head) was not as supportive to SHGs as in other villages. Only once intralending was done so there was no benefit from interest to the group. They broke up because of disagreement among the members regarding the deduction of rupees 10 from each of them to cover the petrol/food expenses of the person depositing the money in the bank. Many people travelled to find work and could not continue depositing money in the group. Group members gradually stopped depositing money and stopped meeting up. The saved money is still in the bank; however, many of the older members now have joined other groups.

#### SHG D: the family-run group

This group was established in 2017 and had 16 members from the start until the time of interview. They appeared to be part of an earlier group that was resurrected during the SHCDP activities. The secretary was a person affected by leprosy and a Mitanin (health worker) and the group leader had a disability. Group leader, secretary and treasurer were related to each other and were quite outspoken and ambitious. The group members appeared to respect the leaders and were satisfied with their methods. They appeared united in their decision-making and helped each other deposit money when members migrated for work temporarily. Their families seemed supportive, and their men seemed to be quite involved in activities. They registered their group in 2021, stating that the delay was due to money shortage. They were yet to create a bank account and were comfortable with keeping the money in their homes. They have done intralending, and multiple loans have been taken by group members over the years. They had some livelihood training, but they stated that since people kept migrating to find work outside the village, they could not start any economic activities. Inability to pay bribes to get into government schemes had also hindered them from getting work. However, they have engaged in plenty of social activities like village cleaning, health camps and petitioning for school and transport facilities. After the project support ended, they continued to deposit rupees 20 monthly (with a late fee of rupees 5) and met on the first of every month.

#### SHG E: the namesake group

The group formed in 2013. It initially had 12–13 members, but it dropped down to 8–9 members because of a few deaths and 2 members leaving the group stating lack of benefit. The group had a bank account and were registered with the government. Initially, they met together every 15 days but by the time of the interview they had stopped meeting many years ago. The group leader appeared to be illiterate and content with focusing on her farming activities and allowing the secretary to run the group; male family members also were involved in many group activities (going to the bank, maintaining records, etc). It appears that they had no training for livelihood. They were initially depositing 20 rupees every eighth day for almost a year, but this proved too much for members to pay, and they decided to stop depositing. No internal lending was carried out. They were able to access the midday meal government scheme most likely with the help of the previous village head who was the group secretary’s brother. After 1 year of passing around the midday meal programme to different members and their families, the group secretary took control of the scheme and others have not received their turns. A few group members felt undervalued because they could not contribute due to their illiteracy and wanted to leave the group. They also resented the lack of transparency from the leaders. One of the members described it as a ‘namesake’ group.

### Drivers of sustainability in self-help groups

Our findings indicate that sustainability of SHGs is underpinned by three core drivers: leadership and governance, economic performance and social performance.

- The first factor, leadership and governance, was based on the internal structure of SHG that guided its operations and maintained accountability. Its features were democratic, responsive and motivated leadership, transparent and flexible governance with regular meetings and support from family and outsiders. SHG participation facilitated disability self-care, and social reintegration for persons affected by leprosy, consistent with chronic infectious disease empowerment models.

The second driving factor we identified was economic performance which reflected the SHG’s financial health, livelihood activities and its ability to provide economic benefits to members. Its core features included consistent collection of membership fees with flexibility for persons with varying economic backgrounds, an internal lending system, including loans to outsiders with interest and enhancing capital growth, equitable distribution of profits, increasing member motivation and satisfaction, access to livelihood opportunities and skill-building programmes, linkages with government schemes and income-generating activities supported by proper financial documentation and functioning accounts.

Social performance was the third driver that encompassed how SHGs contribute to the social empowerment of members and community transformation. Its features included being recognised and respected within the village. SHGs played a critical role in reducing stigma around leprosy and disability through awareness and inclusion. Social and emotional bonds, along with economic benefits, encouraged long-term membership. There was a strong sense of unity and shared mission among members.

When leadership and governance, economic and social performance were strong and balanced, the SHGs remained functional and active over time, adapting to challenges and opportunities to achieve both economic independence and social transformation.

We analysed the five SHGs using the three drivers of sustainability. SHGs A, B and D were found to be sustained, SHG E was non-functional in practice, and SHG C had discontinued.

### Leadership and governance

#### Effective leadership

The leadership style of the SHGs was diverse, which impacted their overall effectiveness. Strong leadership, as seen in SHGs A and B, led to transparency, trust and engagement, creating unity and resilience within the groups. SHG D showed that family-run governance can be effective when leaders prioritise inclusion and group welfare. Challenges arose in SHG C, where leadership struggles led to division, and SHG E, where financial mismanagement eroded trust.

The problem arises when president and secretary hide information of money. They should not be hiding any information, instead it should be shared among everyone in the group. SHG member SHG C—Insider.

#### Flexibility in group arrangements

Regular information about where and when to meet was beneficial to keep the SHGs thriving. In SHG D internal migration to find work hindered attendance, engagement and livelihood activities. During the pandemic, the members of SHG A and B met by phone and ensured well-being of each other. They also used phone calls to plan and implement relief activities for households affected by the pandemic.

…. they take it (decision) immediately by calling each other through mobile phones. So, something good has happened here. Community development officer—SHCDP (Insider).

Setting up a savings and intralending scheme was important in binding group members together in a common purpose. Successful groups SHG A and D waived late fees for members facing financial challenges, especially women affected by leprosy and disability; this enabled them to continue as group members. Groups also allowed late fee payments with minimal fines providing grace period for members unable to pay their contributions immediately, showing a commitment to mutual support and reinforcing group solidarity. Financial assistance was provided to fellow members for important life events like marriages and funerals. This contributed to a family-like environment, supporting and assisting one another both within and outside the group even though the members belonged to different social strata.

We are saving money in the group and helping poor people. We are not forcing members like other groups. Some groups charge a ‘late’ fee and all other things which makes group members irritated. These things are not there in our group. We just wanted to keep the group alive. If members are not able to come, then they can send any other family member from home. Like this, we are keeping our group alive.—SHG member, SHG A—Insider.

#### Family support for SHG activities

In most SHGs, family members contributed to the success of the groups in various ways. They assisted in tasks like buying raw materials, maintaining accounts, doing bank-related work and accompanying group members when needed. This could potentially be beneficial for leprosy-affected women. Some families (as in group B) were unconvinced of the abilities of the women at first but later saw the benefits and supported them fully.

Our son fills/writes the register, or the president writes on the register. Did you open an account with the help of your husband?: Yes, in the beginning he helped us.—SHG C member—Insider.

Where male family members became dominant, as with SHG E, women’s empowerment within the SHGs was blocked.

The group was formed by women and for women, but men started handling the group. If they go to the bank, all work is done by men and if asked by the bank person where is president and others, they show the women and get the sign done. Because of this, they don’t get self-confidence, and because of this the group collapsed.—Sarpanch E—Outsider.

### Economic performance

#### Intralending and loans to members

One of the primary reasons women joined SHGs was the intralending in the group, that is, pooling money and using it whenever someone needed a loan. This helped them to save money and provided access to funds at a low interest rate in times of need without having to provide a collateral or security deposits while avoiding high interest repayment rates which are the norm when borrowing from outsiders. They described this as freedom from the clutches of the moneylenders. This preference of internal borrowing reflected trust and support within the SHG community. Therefore, intralending was a major reason why groups stayed together, even if other economic activities (livelihood schemes) did not work.

[Intralending] it’s helpful madam. We can get it (money) from the group but if we go out…… then we must search and ask many people…then I have to give something in place of money…If we are with the group then we don’t have to keep those things for the security deposit…If we take it from outside then it is given with 5-10% interest.—Person affected by leprosy. SHG A—Insider.

One of the virtues that generally stood out was the financial discipline of the successful groups.

#### Income generation through economic activities

All the SHGs had variable exposure to training through government-run programmes for economic activities which were facilitated by the donor organisation during the funded period such as mushroom farming or phenyl making. This training, however, did not include leadership skills or promote transparency and fairness among members, nor were they taught operational risks and mitigation methods. For example, group B lost money and group C engaged in a range of short-term livelihood activities, including fish farming, sack production and Gerua (a reddish-orange dye) production which did not yield much profit. SHG C members alleged that they did not pursue any training after the funding ceased, blaming the leader for this lapse:

But they (leaders) have not given any guidance to the groups of people who have joined them. They are not functioning, even after six months. Because of no guidance or training. After the project was over. Community volunteer—Insider,

Lack of training and economic success discouraged the groups, as they suffered financial loss. Repeated setbacks could result in an increased focus on intralending as a safer income alternative. The persistence of SHG A stood out because despite failures and getting cheated, the group continued to explore new ventures.

[Group B] lost money on incense making when the trainer abandoned them, and their mushroom cultivation project failed due to poor yields. Program manager, Funding agency—Insider.

One of the important ways to earn a livelihood as a group was to link up with a government scheme. This was a tedious process of paperwork often involving letters of recommendation from village heads and potentially bribery. All SHGs faced challenges to access government schemes and benefits. SHG A tried to access the Gauthan scheme (community cattle-rearing), but they faced local obstacles in getting permissions. SHGs B and D cited barriers to accessing schemes due to favouritism, preventing them from accessing potential government support. SHG C, B and G did not find favour with their village heads, so they lost opportunities to access Government schemes. In contrast, SHG A and one member from SHG E received some benefit from good relationships with the village headman.

[Village head] told us that whoever wants to work for Rojgar Guarantee work [a rural employment scheme] can give their photos. He stamped all the photos on the cards.

### Social performance

#### Stigma reduction

An important aim of the promoter organisation was to bring together people with and without leprosy, to reduce the stigma of leprosy and to lift the poorer affected women socially and financially through inclusion with economically better off groups (mixed membership SHGs). This worked well in some of the groups:

What is the reason behind the group’s functioning till now? ……it’s just because of faith in each other. We do not have discrimination like someone is from lower caste or upper caste. We do not have this kind of thing. We are the same, united and will be same in the future and run the group. SHG member A—Insider.

This (SHGs) has resolved both the financial problem and untouchability (discrimination) issue in the village- like they should not be touched, nothing should be eaten from their hand, or no one should sit with them. …. Sitting all together in the group and sharing items among themselves has resolved this issue. They developed self-confidence and felt that they can also live a normal life. (Outsider) Sarpanch E (village head).

#### Benefit for the larger community

SHGs also engaged in wider health-related initiatives, including awareness campaigns, access to healthcare and support for members dealing with the medical aspects of leprosy or other disabilities. Health and well-being was an important theme for some of the groups (such as A, B and D), distinguishing these groups from others that might focus more on economic activities (such as C and E). Some groups also coalesced around wider social issues:

SHG A campaigned for liquor prohibition and a ban on tobacco products, distributed supplements, monitored children’s diets, and promoted sanitation activities. Group leader SHG A—Insider and community development officer (SHCDP)—Insider.

SHGs also upgraded local infrastructure, such as contributing to constructing water pumps, road repairs and cleanliness of the village. By actively engaging in community development, the SHGs became visible and valued ‘*which could open doors to opportunities, resources, and external support, probably contributing to the better functioning’. Bank manager—Outsider.*

This responsiveness to local needs increased the likelihood of sustained community support and ensured the SHG’s long-term viability.

We go ourselves and take responsibility. Here no health camp was organized so we asked that our village also needs a health camp; people who cannot go far for a health check-up will get a benefit from that. We are asking to open a government hospital here also.—Person affected by Leprosy—SHG D Secretary—Insider.

Groups also helped poor members in their villages to receive government amenities like PDS (ration or public distribution system) cards, kerosene and pensions. SHG A distributed groceries to the villagers during the pandemic and lent money for a member’s daughter’s wedding.

#### Personal development

Through increased individual empowerment and skills, members enhanced their self-worth and contributed more effectively to group goals balancing household, farming and SHG duties, which collectively strengthened the group’s resilience. Most of the outsiders commented on the visible development of the SHG members:

… many SHG women have got a lot of recognition, value, and purpose in what they do as SHG members—Assistant Block Development Officer—Outsider.

Where economic ventures were successful, they allowed women to assert control over financial decisions to contribute significantly to household expenses, including education for their children.

In that group, one leprosy-affected woman was there. She has done so many things in her home after taking a loan from the group which was otherwise impossible in her lifetime. She has bought TV, gold and taken her kids out from Government school and put them in private school.— Program manager, Funding agency—Insider.

Improvement could be seen in members’ communication skills. Initially, many members struggled with basic social interactions, but with SHG involvement, they began to engage more confidently and became socially active.

Some people who came in the group were not knowing anything other than wishing ‘Namaste’ … now they have started speaking and asking about our well-being.—Program manager, SHCDP—Insider.

Now we are more confident to talk fluently than earlier…Now if we go to the bank, we can fill out the form and are able to withdraw money*.* Group leader, SHG A—Insider.

This independence reduced members’ reliance on others. Moreover, SHG involvement empowered members to address broader societal issues. For instance, they have stood up against domestic violence by directly intervening and advocating for women’s dignity and equality:

If any drunken man is beating his wife, then this group of women go and tell him that whatever he is doing is wrong. Community leader—Outsider.

## Discussion

This study examined the sustainability of SHGs in a rural, marginalised context in India after withdrawal of external support. Sustainable SHGs demonstrated three interlinked characteristics: transparent leadership, basic financial discipline through savings and intralending, and social cohesion with responsiveness to community needs.

### Leadership and governance

Leadership emerged as the central factor influencing long-term sustainability. SHGs A, B and D benefited from transparent, responsible and collective leadership practices, whereas SHGs C and E experienced mismanagement of funds, unauthorised withdrawals and lack of transparency, ultimately resulting in group disintegration. This aligns with prior studies showing that leadership disputes and lack of trust can cause groups to collapse or break.^36^ Transparency and fairness are critical unifying factors, and when compromised, trust erodes and withdrawal or dissolution becomes likely.^37^ We find the importance of accessible records, accountability mechanisms and structured leadership development within SHG programmes.

Regular meetings and good governance practices—transparent accounting, flexibility in savings contributions and timely loan repayments—also supported sustained group activity.^37^ Fees, fines and repayment structures regulated participation,^38^ but flexibility for poorer members in savings deposits reduced barriers and prevented attrition.^27^ Rigid rules contributed to member dissatisfaction and dropout, particularly in settings with high economic migration.^27 38^

### Financial practices and mutual support

All groups relied on intralending to meet household needs, support children’s education and enhance women’s decision-making power. While livelihood activities and bank linkages were largely unsuccessful, groups continued functioning through internally generated funds. This supports evidence that financial performance depends on collective savings and prudent credit practices.^38^ Sustained intralending beyond project support reflects trust and mutual responsibility, reducing dependence on informal moneylenders and debt cycles.^37^^,^

### Social capital, inclusion and empowerment

Consistent with social capital theory, both bonding capital (trust and support within the group) and bridging capital (external linkages with local authorities, banks and community structures) contributed to group resilience.^39^ Mixed-membership groups—including women affected by leprosy or disability—strengthened inclusion and reduced stigma, contradicting assumptions that socioeconomic diversity fuels conflict.^35^ These findings resonate with WHO’s community-based rehabilitation principles, which emphasise empowerment, social inclusion and livelihood participation for people with disabilities and chronic diseases.^39^

Membership strengthened women’s autonomy and household status by improving access to savings and credit, enabling social and economic participation.^39^ Initially, male family members contributed to women’s mobility and empowerment; however, in one group male takeover of decision-making undermined women’s autonomy. Sustained gender-sensitive support and role clarity are therefore essential.

SHGs A, B and D also led meaningful community initiatives—addressing stigma, sanitation, domestic violence and local infrastructure. Such visible engagement enhanced community respect and reinforced sustainability, consistent with evidence that women’s collectives facilitate social integration and public participation.

### Sustainability perspective

According to Scheirer, sustainability involves the continuation of core programme components aligned with original goals.^40^ SHGs A, B and D retained alignment with SHCDP objectives, whereas SHGs C and E diverged from their social and financial goals. This highlights the importance of embedding leadership capacity, accountability and community linkages during implementation—beyond livelihood skills alone.

### Strengths and limitations

This qualitative study draws on lived experiences of SHG members and stakeholders, providing rich insights into mechanisms of sustainability. While the sample was small, it is comparable to other exploratory studies. Interviews conducted over 2 years after project completion may have introduced recall limitations, and dormant groups showed hesitancy in discussing challenges. Income-expenditure data were not collected; future studies should incorporate household-level economic assessments. Social desirability bias is possible due to NGO affiliation, although reflexive practices and coding triangulation were employed to mitigate this.

## Conclusions and recommendations

This study indicates that SHGs can be sustainable when there is good quality of leadership, financial performance is adequate and social capital is increased. When planning the development of SHGs, a focus on capacity building for leadership is essential. This includes soft skills such as keeping the group together, being a good listener and including everyone in decision making. Internal monitoring mechanisms also ensure sustainability. Access to government grants and bank linkages can be a challenge and policies need to be in place for the underprivileged to be able to overcome them. Innovative and needs-based economic activities should be explored and marketing of products should also be included in the skills training. Success stories of community development add to credibility and can attract funds from corporate social responsibility agencies and NABARD that SHGs need to continue their financial activities, thus promoting sustainability.

It is important that capacity building for sustainability (as stated above) is incorporated when designing SHG programmes. This will attract the funding and motivate the beneficiaries to continue the activities beyond the funding period.

SHGs can sustain themselves beyond external funding when leadership is strong, governance is transparent and social capital is actively developed. Thus, the supporting organisation should (1) prioritise leadership development through soft skills training, (2) establish internal monitoring mechanisms, (3) provide policy support for accessing government schemes and bank linkages and (4) design innovative economic activities with integrated marketing plans, promoting success stories to attract further support.

Incorporating these strategies in SHG design will increase programme appeal to funders and inspire sustained engagement by beneficiaries.

## Supplementary material

10.1136/bmjopen-2025-110417 online supplemental table 1

10.1136/bmjopen-2025-110417 online supplemental file 1

## Data Availability

Data are available upon reasonable request.
