# Supplementary material for: Sustaining community self-help groups beyond donor support: lessons from a qualitative study of self-help groups, including persons affected by leprosy and disability in rural India
Source: BMJ Open. 2026 Jan 9;16(1):e110417. doi: 10.1136/bmjopen-2025-110417 (PMC12815117; doi:10.1136/bmjopen-2025-110417)
Supplement: online supplemental table 1 [file bmjopen-16-1-s001.docx]

### **Appendix 1 :**

### **Table S1 : Comparison of SHG (Self-Help Group) profile**

| Parameter | SHG A: The Enterprising Group | SHG B: The Functional Group | SHG C: The Inactive Group | SHG D: The Family-run Group | SHG E: The Namesake Group |
| --- | --- | --- | --- | --- | --- |
| Years Active | 5–6 years before 2018; active thereafter | 6–7 years  Active thereafter | Active for 4–5 years, now inactive | Active for 5 years | 9 years, but inactive for many years |
| Initial & Current Members | Started with 19; retained all | Started with 20; reduced to 10 | Started with 13; now inactive | 16 members consistently | Started with 12–13; now 8–9 |
| Group Formation | Members from different SHGs with prior experience | Self-initiated after observing others | Formed but lost direction quickly | Reorganized from an earlier group | Formed with government registration and scheme access |
| Savings Practices | Rs 50/month; consistent savings | Rs 50, later Rs 100/month; regular deposits | Deposited once; discontinued | Rs 20/month; continues even after support ended | Rs 20 every 8th day for a year; then stopped |
| Intra-lending | Yes, at 2% interest | Yes; shared bank interest yearly | Done only once | Regular intra-lending | None |
| Livelihood Activities | Yes; some success; 8 members in midday meal scheme | Yes; 2 shops (grocery/vegetable) | No livelihood | No activity started due to migration and lack of funds | Midday meal handled by one family; no real group livelihood |
| Training Received | Livelihood and leprosy awareness | Livelihood training | No training | Some livelihood training; no implementation | Only initial introduction |
| Group Dynamics & Leadership | Secretary had leprosy and was illiterate; group united, proactive in social causes | Secretary educated and managed activities; group consultative and partially active | Poor leadership, internal disputes led to disbanding | Secretary (leprosy-affected Mitanin); leader with handicap; respected leadership | Secretary’s family controls; leader disengaged; resentment over transparency |
| Family & Community Support | Strong family support; socially proactive | Supportive families: group helped during demonetization | Migration and lack of cohesion caused breakdown | Strong family involvement: men involved; support for each other | Some male involvement; partial support; some members felt undervalued |
| Social Activities | Yes – women’s rights, closing liquor shops, leprosy awareness, charity | None | None | Yes – village cleaning, health camps, petitions for services | Very limited or none |
| Bank Account & Registration | Yes | Yes | Yes | Registered recently; no bank account yet | Yes |
| Access to Govt. Schemes | Ineligible due to large group size | No access due to biased village leadership | None | Could not afford bribes; no schemes received | Midday meal scheme accessed through connections |
| Current Status | Active and willing to work if opportunities arise | Active; united but divided in interest toward livelihood | Inactive | Active, despite migration and no external support | Inactive; name-only group |
