# Supplementary material for: Sustaining community self-help groups beyond donor support: lessons from a qualitative study of self-help groups, including persons affected by leprosy and disability in rural India
Source: BMJ Open. 2026 Jan 9;16(1):e110417. doi: 10.1136/bmjopen-2025-110417 (PMC12815117; doi:10.1136/bmjopen-2025-110417)
Supplement: online supplemental file 1 [file bmjopen-16-1-s002.docx]

### **Appendix 2 : Research team**

The research team comprised full-time researchers with specialized training in qualitative research methods consisting of Principal Investigator (JD), CO Principal Investigator (KG) , Program Manager (AS) , Research officers (MV and TL) and research assistants (RH, TH). Dr JD, PI is a family physician and the head of research domain of the leprosy mission trust India and , Mr KG , Co PI is a public health expert currently pursuing PHD in public health. AS, the Program Manager is master’s in public health. The research officers have master's degree and the RA’s hold graduate degrees with training in research interviews. The PI and PM were the 2 female researchers, rest were males.
