# Supplementary material for: Sustaining community self-help groups beyond donor support: lessons from a qualitative study of self-help groups, including persons affected by leprosy and disability in rural India
Source: BMJ Open. 2026 Jan 9;16(1):e110417. doi: 10.1136/bmjopen-2025-110417 (PMC12815117; doi:10.1136/bmjopen-2025-110417)
Supplement: Supplementary file 3 [file bmjopen-2025-110417.reviewer_comments.pdf]

PEER REVIEW HISTORY

BMJ Open publishes all reviews undertaken for accepted manuscripts. Reviewers are asked to complete a checklist review form and are provided with free text boxes to elaborate on their assessment. These free text comments are reproduced below.

ARTICLE DETAILS

Title (Provisional)

Sustaining Community Self-Help Groups Beyond Donor Support: Lessons from a qualitative study of Self-Help Groups, Including Persons Affected by Leprosy and Disability in Rural India

Authors

Darlong, Joydeepa; Charles, Mythily Vandana S; ILOZUMBA, ONAEDO; Govindasamy, Karthikeyan; Shrivastva, Anjali; Choudhury, Sopna; Sartori, Jo; Lindenmeyer, Antje; Lilford, Richard J; Griffiths, Frances

---

VERSION 1 - REVIEW

**Reviewer**	**1**
**Name**	**Fastenau, Anil**
**Affiliation**	**German Leprosy and Tuberculosis Relief Association**
**Date**	**15-Oct-2025**
**COI**	**None**

Dear Authors,

Thank you for submitting your manuscript. I have reviewed the paper and have no further comments. I accept the manuscript in its current form.

**Reviewer**	**2**
**Name**	**Magalhães, Lucas**
**Affiliation**	**Universidade Federal de Sergipe**
**Date**	**22-Oct-2025**
**COI**	**None**

Dear authors,

I have reviewed the manuscript and would like to clarify some key points. The paper examines the post-project sustainability of self-help groups (SHGs) of patients affected by leprosy and disabilities in rural India. The topic is relevant and contextually significant, particularly in the areas of community-based rehabilitation and public health related to chronic diseases such as leprosy. However, the study's contribution to global knowledge appears limited.

Major comments:

• The background relies heavily on grey literature and outdated sources. Please incorporate more recent and international studies to enhance the manuscript's global relevance.

• The findings presented in the Results section, focusing on leadership, economics, and social engagement, reiterate existing SHG and microfinance literature without offering new theoretical insights. Please strengthen this section, particularly by relating the findings to infectious diseases.

• The Discussion section fails to connect local results with broader development or health systems frameworks. Furthermore, the thematic analysis is largely descriptive and lacks theoretical grounding, which would better contextualize the findings and support their potential applications in health policy, for example.

• It is also important to include a more thorough discussion of researcher bias and positionality to clarify potential methodological influences.

Minor comments:

• The title is inadequate and does not accurately reflect the manuscript's content, as SHGs are used for various diseases and not only leprosy. Please propose a new title that better fits the scope of the study.

• The subsection structure within the Introduction section is awkward and unnecessary. I suggest revising the introduction to make it more concise and specific to the topic (i.e., SHGs in the context of leprosy and/or infectious diseases).

VERSION 1 - AUTHOR RESPONSE

**1. The background relies heavily on grey literature and outdated sources. Please incorporate more recent and international studies to enhance the manuscript's global relevance.**	**Changes in manuscript: 107-109, 141, and 143-146.**We have incorporated recent global research on SHGs, chronic infectious diseases, disability inclusion, and social protection (2021–2024 studies). We updated references on SHGs in global health, disability empowerment, and microfinance, especially in LMIC settings.

	We added recent studies and updated SHG and disability empowerment literature (2021–2024). We also provide references to SHG sustainability in LMICS<br>(Sondaal AE, Tumbahangphe KM, Neupane R, Manandhar DS, Costello A, Morrison J. Sustainability of community-based women's groups: reflections from a participatory intervention for newborn and maternal health in Nepal. Community development journal. 2019;54(4):731-49.<br><br>Bunning K, Gona JK, Wanjala SW, Hartley S. The Sustainability of Self-Help Groups for Caregivers of Children With Disabilities in Kilifi, Kenya: The Changing Context of the COVID-19 Pandemic. Child Care Health Dev. 2025;51(3):e70079)
**2. The findings presented in the Results section, focusing on leadership, economics, and social engagement, reiterate existing SHG and microfinance literature without offering new theoretical insights. Please strengthen this section, particularly by relating the findings to infectious diseases.**	Infectious disease linkage was added as advised.<br>**Changes in manuscript: 338-341,** 548-553<br>• SHG participation facilitated health-seeking, disability self-care, and social reintegration for persons affected by leprosy, consistent with chronic infectious disease empowerment models<br>• We also pick up this point in the discussion where we included mixed-membership SHGs (with and without leprosy/disability) facilitating stigma reduction.

**3. The Discussion section fails to connect local results with broader development or health systems frameworks. Furthermore, the thematic analysis is largely descriptive and lacks theoretical grounding, which would better contextualize the findings and support their potential applications in health policy, for example.**	We have rewritten the Discussion drawing on social capital theory.
**4. It is also important to include a more thorough discussion of researcher bias and positionality to clarify potential methodological influences.**	Added a subsection on positionality: 216-220<br>• Co-researchers affiliated with NGO may influence responses.<br>• Women researchers and local field staff conducted interviews.<br>• Steps for minimizing bias: COREQ-conform procedures, independent transcription, intercoder reliability, reflexive memos.
**5. The title is inadequate and does not accurately reflect the manuscript's content, as SHGs are used for various diseases and not only leprosy. Please propose a new title that better fits the scope of the study.**	Revised title to reflect broader community context and chronic disability focus.<br>Sustaining Community Self-Help Groups Beyond Donor Support: Lessons from Mixed-Membership Groups Including Persons Affected by Leprosy and Disability in Rural India.
**6. The subsection structure within the Introduction section is awkward and unnecessary. I suggest revising the introduction to make it more concise and specific to the topic (i.e., SHGs in the context of leprosy and/or infectious diseases).**	We removed excess headings, merged background and sustainability of SHGs.

VERSION 2 - REVIEW

**Reviewer**	**2**
**Name**	**Magalhães, Lucas**
**Affiliation**	**Universidade Federal de Sergipe**
**Date**	**17-Nov-2025**
**COI**	

Thank you the revised version.
